# Biogenic Amines as Biomarkers for the Assessment of Diesel-Contaminated Water Toxicity

**DOI:** 10.3390/molecules31162838

**Published:** 2026-08-14

**Authors:** Łukasz Sikorski, Agnieszka Bęś, Wojciech Truszkowski, Amit Kumar, Andrzej Brandyk, Maja Radziemska

**Affiliations:** 1 Department of Chemistry, Faculty of Agriculture and Forestry, University of Warmia and Mazury in Olsztyn, Pl. Łódzki 4, 10-727 Olsztyn, Poland; lukasz.sikorski@uwm.edu.pl (Ł.S.); agnieszka.bes@uwm.edu.pl (A.B.); 2 Department of Agrotechnology and Agribusiness, Faculty of Agriculture and Forestry, University of Warmia and Mazury in Olsztyn, M. Oczapowskiego 8, 10-719 Olsztyn, Poland; wojciech.truszkowski@uwm.edu.pl; 3 School of Hydrology and Water Resources, Nanjing University of Information Science and Technology, Nanjing 210044, China; amitkdah@nuist.edu.cn; 4 Institute of Environmental Engineering, Warsaw University of Life Sciences, Nowoursynowska 159, 02-776 Warsaw, Poland; andrzej_brandyk@sggw.edu.pl

**Keywords:** diesel oil, algae, common duckweed, phytotoxicity, photosynthesis, biogenic amines

## Abstract

This study evaluated the phytotoxicity of diesel oil (DO) using the green alga *Pseudokirchneriella subcapitata* and the aquatic plant *Lemna minor*. Toxic effects were assessed in 7-day Algaltoxkit and Lemna bioassays by measuring growth, chlorophyll fluorescence, and biogenic amine (BA) content. *L. minor* was less sensitive to DO in terms of growth than the alga. Toxicity thresholds (LOEC, 7 days) showed that DO concentrations above 0.11–0.12% significantly inhibited the growth and productivity of both model organisms. Chlorophyll fluorescence proved to be an early and sensitive indicator of DO toxicity. This study also provides the first characterization of BAs in *P. subcapitata*, identifying histamine, tyramine, putrescine, cadaverine, agmatine, spermidine, and spermine. Agmatine emerged as a common indicator of DO contamination in both species. Its content decreased in *P. subcapitata* (EC_50_ = 0.85%) but increased in *L. minor* (EC_50_ = 0.38%) under diesel exposure. The findings herein suggest that BA profiling, especially agmatine, can support early detection of hydrocarbon stress in aquatic organisms.

## 1. Introduction

Freshwater environments (rivers, lakes, ponds, wetlands, and groundwater) are extremely valuable ecosystems, performing biological, hydrological, and retention functions, as well as providing freshwater for humans and animals. Pollution resulting from anthropogenic activities poses a serious threat to the functions of those ecosystems. One such pollutant is fuel, including diesel fuel (DO). Petroleum substances are derived from the distillation of crude oil into various fractions. These fractions are generally classified into four main categories: light distillates (such as liquefied petroleum gas, gasoline, and naphtha), middle distillates (including kerosene and diesel oil), heavy distillates (such as heavy fuel oil, lubricating oils, and paraffin waxes), and residual fractions (primarily asphalt) [1]. Pollution of the environment with oil and petroleum products is a global problem. The source of such pollution is not only accidents of drilling platforms or tankers but also their operation, the surface transport, processing, and storage of fuels [2]. World Oil Outlook forecasts indicate a continuous increase in global demand, which in 2035 will amount to 117.9 million barrels per day of diesel fuel (DO) [3]. Chemically, DO constitutes a complex mixture of hydrocarbons: aliphatic (alkanes, cycloalkanes), aromatic (including polycyclic aromatic hydrocarbons, PAHs), and various auxiliary components and admixtures [4]. It also contains trace amounts of sulfur, nitrogen, and oxygen, as well as metals such as lead, nickel, sodium, calcium, copper, and uranium [5].

Diesel fuel components, especially in the soluble fraction (WSF/WAF), may be toxic to aquatic organisms, i.e., plankton, microalgae, invertebrates, and fish. The toxicity of ON depends on the dose, duration of exposure, and the species of the tested organism [6]. The mean mortality rate of planktonic crustaceans (*Daphnia magna*) exposed to diesel oil is 78.34%, with an acute toxicity LC_50_ value of 1.78 ppm. The rainbow trout (*Oncorhynchus mykiss*) is more resistant, showing a mortality rate of 38.33% at the lowest tested diesel concentration (100 ppm), while the average mortality after 96 h of exposure reaches 70.17%. The LC_50_ value for acute toxicity test using *O. mykiss* is 133.52 ppm [7]. Therefore, it is important to study the ecological effects of this xenobiotic on other freshwater organisms, including plants. Brown algae *Sargassum cymosum* var. stenoplhyllum exposed to DO showed an increase in the thickness of the cell wall, the number of vacuoles in the cells, and the content of chlorophyll a and c, as well as a corresponding decrease in the concentration of phenolic compounds [8]. Meanwhile, water hyacinth *Eichhornia crassipes* exposed to DO for 3 to 14 days showed the following changes: growth inhibition and chlorosis, necrotic spots on the leaf surface, slower plant growth and leaf drop, and increasing stuntedness [9]. As indicated, DO-polluted water changes not only the morphological but also the biochemical features of plants.

In environmental studies, it is important to detect sublethal effects, including the first symptoms of toxicity, as well as sensitive biomarkers from the viewpoint of ecosystem safety. The toxicity of DO could manifest in the form of disturbances in physiological processes, including the inhibition of photosynthesis and, consequently, the emission of fluorescence. Damage to the photosynthetic apparatus, although its symptoms are not yet visible, can cause a decrease in the efficiency of photosynthesis in plants, which results in a decrease in growth rate. Studies have shown that the WSF of DO is toxic to microalgae (e.g., *Pseudokirchneriella subcapitata* and *Chlorella* sp.), causing a decrease in chlorophyll a (a growth parameter) and induction of oxidative stress mechanisms (antioxidant enzymes such as superoxidase dismutase, catalase, and peroxidase) [10]. However, it is not yet known how planktonic and floating plants (one indirectly, the other directly exposed to DO floating on the surface of the medium) respond to DO in qualitative and quantitative changes in the biogenic amines (BAs) contained in them. Biogenic amines are low-molecular-weight organic compounds naturally occurring in plant and animal tissues and in microorganisms. These compounds contain a characteristic amine group (-NH_2_) and are formed primarily through the decarboxylation of amino acids. In plants, they function not only as secondary metabolites but also as important regulators of physiological processes. Biogenic amines participate in numerous biological processes, including the regulation of cell growth and division, tissue differentiation, and responses to abiotic stresses such as drought, low temperatures, nutrient deficiencies, and environmental pollution. Their increased synthesis and accumulation under stressful conditions indicate their key role in adaptive mechanisms. They act as signaling molecules that modulate enzyme activity, cell membrane permeability, and reactive oxygen species (ROS) levels, allowing plants to more effectively counteract oxidative damage. Furthermore, biogenic amines, such as putrescine, spermine, and spermidine, are directly involved in the processes of fruit ripening, flowering, and aging in plants. They regulate the stability of nucleic acids, protect protein structures, and contribute to the maintenance of the integrity of biological membranes. Therefore, they are considered important factors in plant resistance to environmental stress and proper functioning under changing environmental conditions [11].

Despite extensive use of algal and macrophyte bioassays in ecotoxicological evaluations of petroleum-derived contaminants, knowledge of the physiological and biochemical mechanisms underlying plant responses to diesel oil exposure remains limited. In particular, while growth inhibition and chlorophyll fluorescence are commonly applied toxicity endpoints, the role of biogenic amines in mediating or indicating diesel-induced stress in aquatic primary producers has not been sufficiently explored. Data on the biogenic amine profiles of green algae under xenobiotic pressure are especially scarce, and species-specific differences in amine regulation between algae and higher aquatic plants remain poorly understood. Moreover, the enzymatic pathways governing biogenic amine biosynthesis, including the activity of arginine decarboxylase, have not been systematically investigated in the context of diesel oil contamination, limiting the identification of reliable biochemical biomarkers of exposure and effect. Moreover, freshwater microalgae, as test organisms, are typically exposed to the test substance in batch cultures for 72 h [12]. Therefore, to compare the ecological effects of DO across two plant species, the alga *P. subcapitata* and the macrophyte *L. minor*, the exposure duration was standardized to 7 days. This study aimed to assess the phytotoxicity of DO in aquatic environments by evaluating selected morphological and physiological features of the algal species *P. subcapitata* and the plant species *L. minor*. The toxicity of DO was assessed using a 7-day Algaltoxkit and Lemna test by determining the growth of plants as well as the fluorescence activity of chlorophyll and BA content. Despite extensive studies on the phytotoxicity of petroleum hydrocarbons, little is known about the role of biogenic amines in the physiological responses of aquatic primary producers to DO contamination. In particular, the biogenic amine profile of *P. subcapitata* has not been previously characterized, and the potential of these compounds as early biomarkers of DO-induced stress remains largely unexplored.

## 2. Results

### 2.1. Effect of DO on the Ir and Iy of P. subcapitata

**Figure 1:**
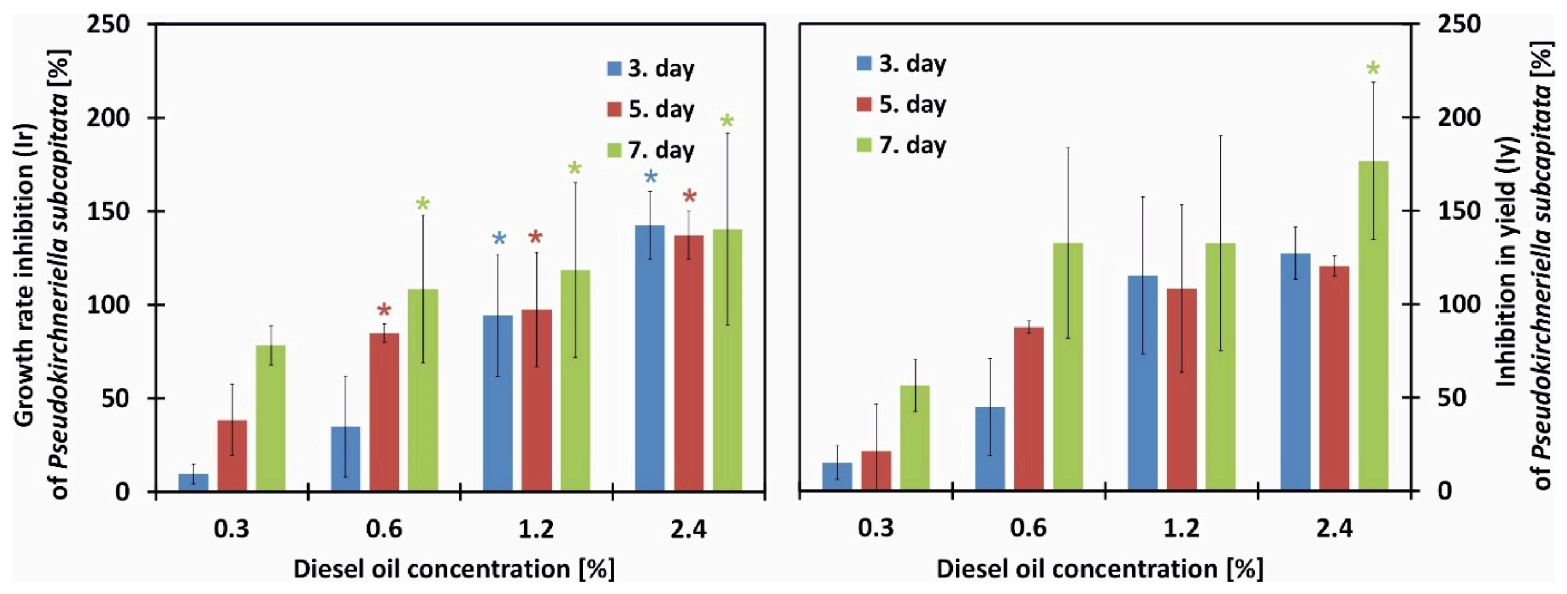
Percent inhibition of growth rate (Ir) and inhibition in yield (Iy) of *P. subcapitata* exposed to different concentrations (0.0–2.4%) of diesel oil (DO). Data points represent the mean ± SD, *n* = 6. * Values differ significantly from the control at *p* < 0.01.

The analysis of variance (ANOVA) revealed that Ir was independently influenced by the concentration of DO and the duration of the test. However, Iy changed only under the influence of DO concentration (Table S1). On day 3 of the experiment, the Ir of algae at the lowest concentration (DO = 0.3%) was 9.66%, and then it increased up to 142.57% at the highest concentration of DO. After five days of algae exposure, Ir also increased with increasing xenobiotic concentrations. However, at concentrations of 0.3 and 0.6% of DO, they were higher, 35.57 and 84.95%, respectively. On day 7, it was the highest, and at a concentration of 0.3% DO, it had a value of 78.41%, while at 0.6–2.4% DO, Ir had an average of 122.45%. Meanwhile, Iy on days 3 and 5 changed similarly to Ir, but this was not statistically significant. On day 7, Iy was the highest, and at concentrations of 0.3, 0.6, 1.2, and 2.4%, it was 56.52, 123.84, 132.71, and significantly 176.69%, respectively (Figure 1).

Confirmation of the toxicity of DO was provided by the effective concentrations EC_20_, EC_50,_ and EC_90_ calculated on days 3, 5, and 7—based on Ir and Iy—inhibiting growth parameters by 20, 50, and 90%, respectively. On day 3, the EC_20_, EC_50,_ and EC_90_ against Ir were 0.54%, 0.71%, and 1.29% DO, respectively. The effective concentrations determined on day 5 for Ir were lower than after 3 days of exposure to DO, reaching EC_20_, EC_50_, and EC_90_ values of 0.08%, 0.19%, and 0.39%, respectively, on the last day of the test. The calculated acute toxicity indices for Iy algae on the 3rd and 5th day of the study were approximately 30% lower than Ir. However, on the 7th day of the study, they (Iy) were slightly higher, with values of 0.14, 0.26, 0.43% for EC_20_, EC_50_, EC_90_, respectively (Table 1).

### 2.2. Effect of DO on the Ir and Iy of L. minor

**Figure 2:**
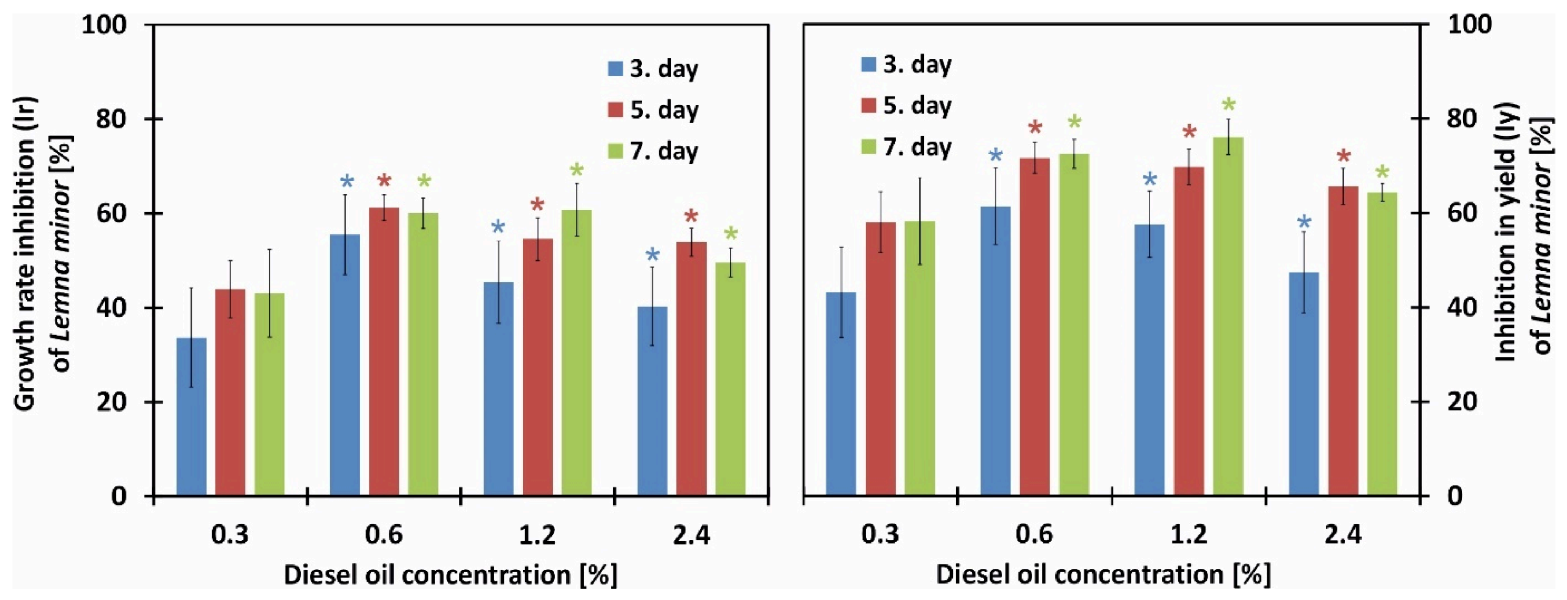
Percent inhibition of growth rate (Ir) and inhibition in yield (Iy) of *L. minor* exposed to different concentrations (0.0–2.4%) of diesel oil (DO). Data points represent the mean ± SD, *n* = 6. * Values differ significantly from the control at *p* < 0.01.

Statistical analysis showed that Ir of *L. minor* was modified by DO concentration, while Iy was influenced by both DO concentration and experimental duration (Table S2). The *L. minor* plants reacted more sensitively to the lowest (0.3%) DO concentration than the algae. Already on day 3, the Ir value was 33.69%, then on days 5 and 7, plant growth was inhibited by approximately 43%. The highest statistically significant Ir values were recorded for the concentration of DO = 0.6%, and on each day, i.e., 3, 5, and 7, it was approximately 58%. On days 3 and 5, duckweed acclimated somewhat to the two highest concentrations (1.2 and 2.4%) of DO, as Ir in these samples was slightly lower. On the final day of exposure, for the same xenobiotic concentrations, the average increase in response (Ir) was significantly 55%. Meanwhile, Iy of *L. minor* was more sensitive because this plant yielded an inhibition parameter that was higher than Ir. Similar to the case of Ir, the Iy index indicates that the plants were able to acclimatize to the highest DO concentration, because Iy at this concentration decreased compared to the previous one (Figure 2).

Confirmation of plant acclimatization in higher concentrations of DO was provided by the calculated EC_x,_ which inhibits plant growth and yield. On no day of the experiment was the Ir or Iy of *L. minor* inhibited by as much as 90%. Also, the EC_20_ and EC_50_ were higher than those recorded for algae *P. subcapitata*. The EC_20_ for Ir on day 3 was 0.20%, while on days 5 and 7, it was the same, at 0.14% (Table 2).

The EC_50_ for the same growth parameter (Ir) on days 3, 5, and 7 was 2 times higher than EC_20_ on average. The EC_20_ for Iy was, on average, 75% lower than those calculated for Ir on each day. Meanwhile, the yield inhibition by 50% on day 3 was at the level of DO = 0.51%, and on days 5 and 7, it was at the level of 0.26% of DO (Table 2).

### 2.3. Effect of DO on the Biochemical Features of P. subcapitata

**Figure 3:**
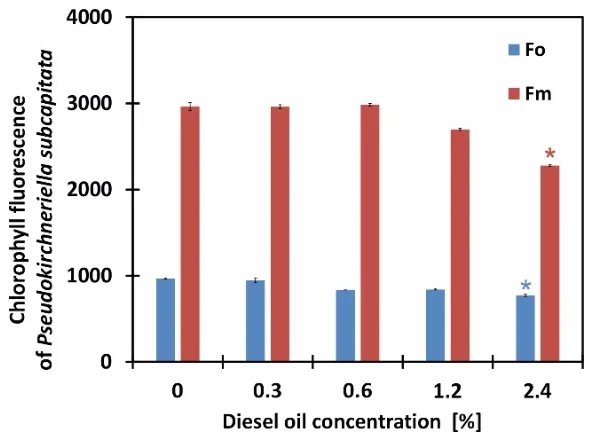
The minimum chlorophyll fluorescence (Fo) and maximum chlorophyll fluorescence (F_m_) of *Pseudokirchneriella subcapitata* exposed to different concentrations (0.0–2.4%) of diesel oil (DO). Data points represent the mean ± SD, *n* = 6. * Values differ significantly from the control at *p* < 0.01.

On the last (7th) day of the test, biochemical analyses were also performed, including photosynthetic activity and BA content. It was shown that Fo (minimum fluorescence after dark adaptation), Fm (maximum fluorescence after dark adaptation), Fv/Fm (variable fluorescence after dark adaptation) were modified by DO concentration (Table S3). The Fo decreased with increasing concentration of this xenobiotic. The highest Fo value (969.33) was recorded in the control sample, and DO concentrations of 0.3 M and 2.4% inhibited Fo by 13.86% and significantly by 20.26%, respectively. The lowest concentrations of DO (0.3 and 0.6%) did not reduce the Fm of algae, which was Fm = 2973, similar to the control sample. Thereafter, the value of Fm decreased. The DO = 1.2% reduced Fm by 9%, and DO = 2.4% reduced Fm significantly by 23% compared to the control sample (Figure 3).

The DO toxicity was reflected in Fv/Fm measurements, which are the most sensitive indicator of photochemical activity of the photosynthetic apparatus. Although the index increase from 0.66 in the control sample to an average of 0.68 in samples containing 0.3–2.4% of DO was statistically significant, the magnitude of the change was small (Table 3). In this experiment, the BA profile in the cells of the green alga *P. subcapitata* was assessed for the first time. The analysis confirmed the presence of several types of BAs within the plant tissues, including monoamines such as histamine (His) and tyramine (Tyr); diamines such as putrescine (Put), cadaverine (Cad), and agmatine (Agm); and the triamine spermidine (Spd) and the tetraamine spermine (Spm). Statistical analysis using an ANOVA indicated that the contents of these BAs varied significantly depending on the DO concentration (Table S3). Histamine in the control sample was found in 14.34 µg g^−1^ of tissue. Increasing DO concentrations caused a significant reduction in the amount of this amine, and at a concentration of 2.4% of DO, there was half as much of it as in the control. In the control sample, there was 6.49 µg g^−1^ of Tyr in *P. subcapitata* tissue. Meanwhile, the Tyr content increased to concentrations of 0.3 and 0.6% of DO. At these concentrations, Tyr increased significantly to 15.23 and 11.07 µg g^−1^ of tissue. At higher concentrations of DO, the Put content was similar to the control. The Put content changed somewhat similarly. The control sample contained 6.79 µg g^−1^ of tissue, with the highest concentration of Put being 18.82 µg g^−1^ of tissue in the 0.3% DO medium.

Subsequently, the amount of this BA gradually decreased to 11.12 µg g^−1^ of tissue. Meanwhile, changes in Cad content decreased significantly with increasing DO content in the samples.

**Figure 4:**
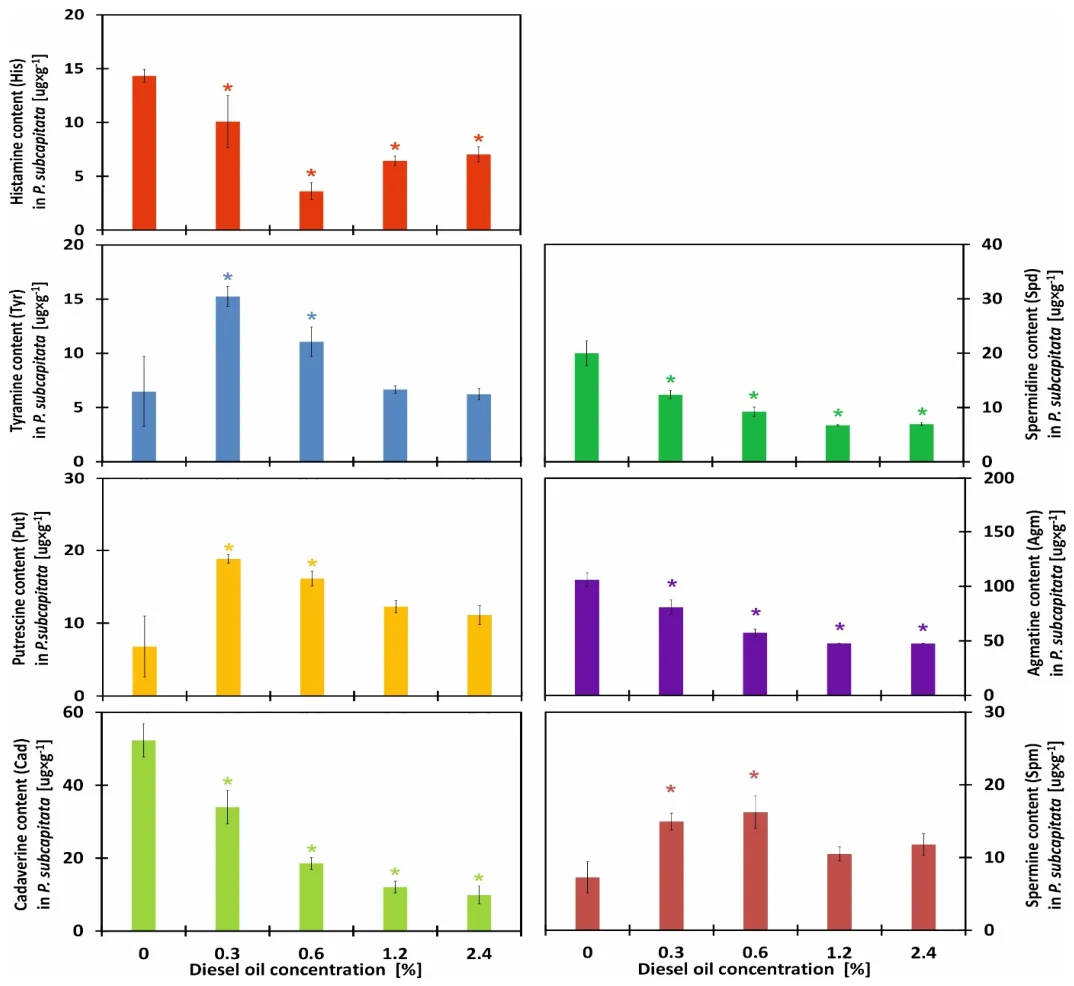
Biogenic amine content (BA) of *Pseudokirchneriella subcapitata* exposed to different concentrations (0.0–2.4%) of diesel oil (DO). Data points represent the mean ± SD, *n* = 6. * Values differ significantly from the control at *p* < 0.01.

The control sample contained 52.33 µg g^−1^ of Cad, while those from contaminated samples contained progressively less Cad, and at a concentration of 2.4% ON, the content was only 9.89 µg g^−1^ of tissue. Diesel oil also significantly influenced the biosynthesis of Spm. Algae not exposed to the xenobiotic had Spm of 19.99 µg g^−1^. With concentrations of 0.2–2.4% of DO, it decreased from 12.37 to 6.96 µg g^−1^ of tissue, respectively. Of all biogenic amines, Agm was the most abundant in algal tissues. Control plants contained as much as 105.96 µg g^−1^ of tissue. However, the DO inhibited the biosynthesis of this compound, and its amount decreased in the samples. The highest DO concentration already contained 47.67 µg g^−1^ of Agm. The highest Spm content was found when plants were exposed to 0.6% of DO, with the control containing 7.28 µg g^−1^ of tissue (Figure 4).

It was shown that among the algal chlorophyll fluorescence indices, only for Fo and Fm, it was possible to determine the concentration inhibiting this physiological reaction by 20%; values of 2.30 and 2.14%, respectively, were found. The most sensitive BA to DO was Put, whose content changed by 20, 50, and 90% at concentrations of 0.03, 0.08, and 0.15%, respectively. Tyr and Spm were slightly less sensitive, but the same toxicity rates were slightly higher for these amines. The calculated His, Cad, Spd, and Agm EC included only changes of 20 and 50% (Table 4).

### 2.4. Effect of DO on the Biochemical Features of L. minor

**Figure 5:**
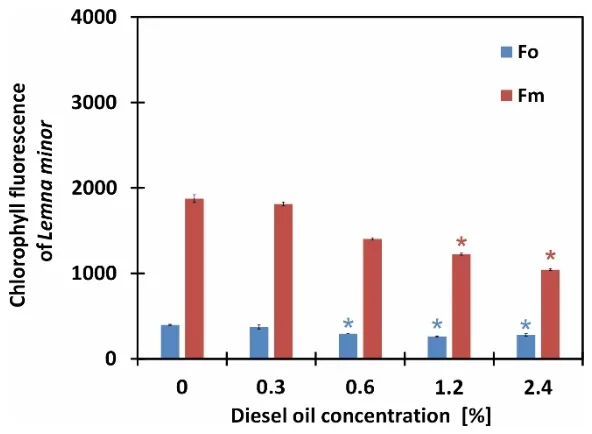
The minimum chlorophyll fluorescence (Fo) and maximum chlorophyll fluorescence (F_m_) of *Lemna minor* exposed to different concentrations (0.0–2.4%) of diesel oil (DO). Data points represent the mean ± SD, *n* = 6. * Values differ significantly from the control at *p* < 0.01.

The chlorophyll fluorescence indices and BA content were also examined in *L. minor* tissues. LCC was also determined in the leaves of these plants. Statistical analysis (ANOVA) showed that Fo, Fm, LCC, and among BAs only Agm changed as a result of DO concentration (Table S4). The experiment examining the effect of DO on *L. minor* showed that Fo decreased as a result of xenobiotic toxicity, with the lowest value of this fluorescence index, 263.33, recorded at a concentration of 1.2% of DO. The statistically significant value recorded at this concentration was 30% lower than in the control. The Fm also decreased when plants were exposed to DO. In the control sample, it was 1875.33, while in the highest concentration (DO = 2.4%), it was only 1043.33 (Figure 5).

**Figure 6:**
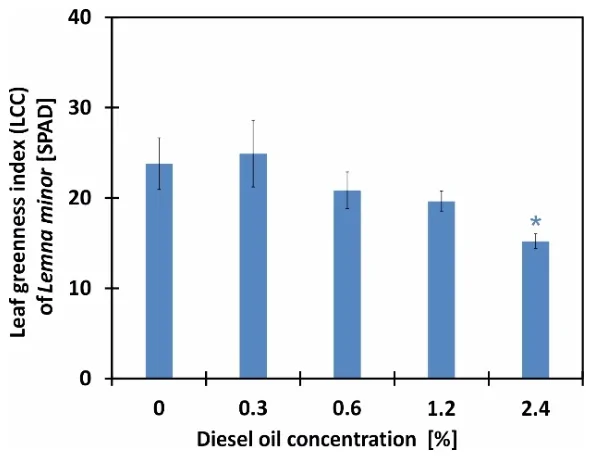
The leaf greenness index (LCC) of *Lemna minor* exposed to different concentrations (0.0–2.4%) of diesel oil (DO). Data points represent the mean ± SD, *n* = 6. * Values differ significantly from the control at *p* < 0.01.

The decrease in photosynthetic indices was the result of increasingly lower LCC, plants. Moreover, while LCC increased by some percentage (24.92 SPAD) in the lowest DO concentration, the highest one decreased to 15.22 SPAD (Figure 6).

The 2.4% of DO in the medium reduced the Fv/Fm to 0.73 from 0.79 in the control plants. However, this change was not statistically significant (Table 5).

The analysis revealed the presence of several BAs in *L. minor* tissues, including monoamines such as Tyr; diamines like Cad, Put, and Agm; the triamine Spd; and the tetraamine Spm. At concentrations of 0–0.6% of DO, an average of 2.17 µg g^−1^ of tissue of Tyr was analysed, while at 1.2–24% of DO, the content of this amine was twice as high. However, the difference was not statistically significant.

**Figure 7:**
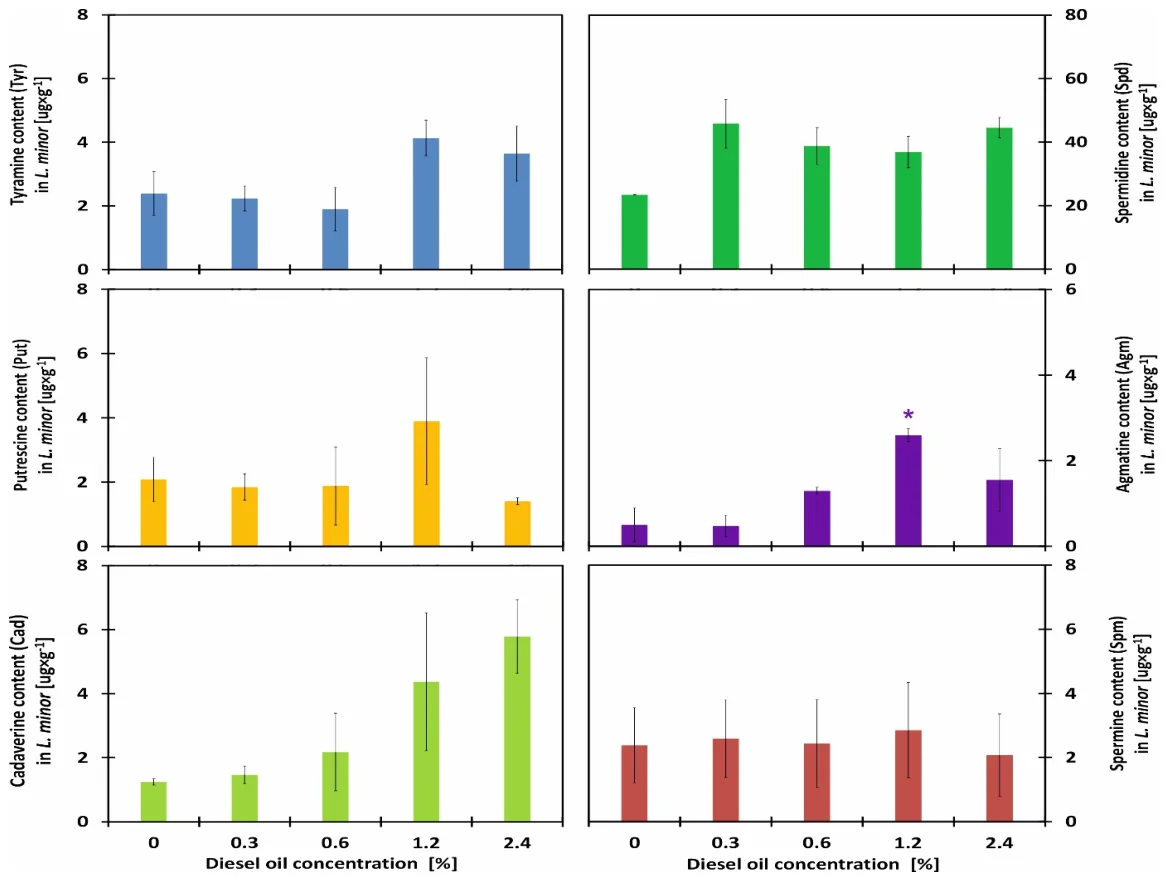
The biogenic amine content (BAs) of *Lemna minor* exposed to different concentrations (0.0–2.4%) of diesel oil (DO). Data points represent the mean ± SD, *n* = 6. * Values differ significantly from the control at *p* < 0.01.

Meanwhile, the plants responded non-significantly to the change in Put content only at a concentration of 1.2% of DO, where 3.90 17 µg g^−1^ of tissue was detected, representing an 86% increase compared to the *L. minor* control. The Cad content increased in direct proportion to the DO concentration in the medium. In the control sample, it was 1.25 µg g^−1^ of tissue, while in the highest concentration (2.4% of DO), it was 5.78 µg g^−1^ of tissue. The observed trend for Cad was not statistically significant, similarly to Spd. The plants reacted slightly differently to the Spd content. The control sample contained 23.45 µg g^−1^ of tissue of this amine, while the contaminated samples contained an average of 41.50 µg g^−1^ of tissue. Plants not exposed to DO contained very small amounts of Agm = 0.44 µg g^−1^ of tissue, but the content of this amine increased significantly at a concentration of 1.2% of DO. In this sample, 2.60 µg g^−1^ of tissue of Agm was recorded. The Spm content did not change as a result of DO. In all samples, it averaged 2.47 µg g^−1^ of tissue (Figure 7).

As indicated by acute toxicity indices, duckweed had 20% less chlorophyll when exposed to 1% of DO. Consequently, these changes induced chlorophyll fluorescence inhibition (EC_20_) for Fo and Fm at concentrations of 0.55% and 0.43% of DO, respectively. The DO had the greatest effect on Spd biosynthesis, as already at concentrations of 0.07, 0.19, and 0.24% (of DO), it increased its content by 20, 50, and 90%. On average, more than 2.5 times more DO was required to accumulate Agm in amounts 20, 50, and 90% higher than in control tissues. Somewhat similarly to Agm, *L. minor* reacted with Cad content. Tyramine and Put changed by 20% and 50% at concentrations of 0.85 and 0.95% DO, on average, compared to the control (Table 6).

## 3. Discussion

Diesel oil and petroleum-derived substances are among the most common environmental pollutants in highly industrialized countries. Their prevalence stems from the extensive use of fossil fuels in transportation, energy, and the chemical industry, leading to a constant risk of emissions and leaks into terrestrial and aquatic ecosystems. These pollutants can occur both in the form of major accidents, such as tanker disasters or pipeline leaks, and in the form of chronic, difficult-to-detect point or diffuse discharges, resulting from, for example, heavy road traffic or machinery operation. Due to the persistence of some petroleum-derived components and their ability to bioaccumulate, they pose a serious threat to plants, animals, and the functioning of entire ecosystems. The introduction of diesel fuel into the aquatic environment can lead to significant changes in its physical and chemical properties, such as turbidity, reduced light penetration, pH changes, and impaired gas exchange. These changes can directly impact the functioning of aquatic plants, limiting photosynthesis and impeding the uptake of essential nutrients. In our study, both low and high DO concentrations were used to create a model covering the impact of the tested toxicant both at environmental concentrations and those associated with accidents and ecological disasters caused by uncontrolled fuel spills. The results of this study indicate that diesel fuel hurts the growth and health of both the algae *P. subcapitata* and the lesser duckweed *L. minor*. Plants exposed to this type of fuel exhibited not only inhibition of morphological traits (growth and tissue degradation) but also biochemical traits (chlorophyll fluorescence and changes in BA content). Comparison of the responses of the two plant types to the tested xenobiotic revealed that DO had distinct toxic effects on algae and floating plants and induced different morphological and physiological changes. On day 3 of the experiment, the EC_20_, EC_50,_ and EC_90_ against Ir of *P. subcapitata* were 0.54%, 0.71%, and 1.29% DO, respectively. The calculated acute toxicity indices for Iy of algae on the 3rd day of the study were approximately lower than Ir. The EC_20_ index on that day was 0.25%. (Table 1). According to Efroymson [13], the lowest observed effect concentration (LOEC) is defined as the concentration of a chemical substance at which the measured adverse organismal response exceeds 20% relative to the control. The EC_50_ values may vary depending on the sample type (crude diesel, WSF soluble fraction, emulsion, etc.), exposure time, and test conditions. The Shell plc (6304) study reported a 72 h ErL_50_ (effect loading rate causing 50% of aquatic organisms) value of 22 mg/L (2.2%) for DO. Likewise, Clark Brands LLC [14] obtained a similar 72 h effect level (ErL_50_ = 22 mg/L) when testing ultra-low sulphur diesel. The Shell plc (6304) study was selected to represent this endpoint as it is a reliable (category 2), GLP-compliant (Good Laboratory Practice), and near-guideline study with no significant limitations. Therefore, it is considered fully adequate for environmental risk assessment [14]. Reduction in plant biomass yield is a common effect of DO and increases with increasing environmental pollution [15,16].

*L. minor* also had impaired growth, including growth inhibition when the medium was contaminated with DO. The plants reacted more sensitively to the lowest (0.3%) DO concentration than the algae. However, the EC_20_ and EC_50_ were higher than those recorded for algae *P. subcapitata*. The EC_20_ for Ir on day 3 was 0.20%, while on days 5 and 7, it was the same, at 0.14% of DO. The EC_50_ for the same growth parameter (Ir) on days 3, 5, and 7 was two times higher than EC_20_ on average (Table 2). It should be noted that *L. minor* was generally less sensitive to the tested xenobiotic concentrations in relation to its growth characteristics. However, toxicity tests for DO conducted equally on duckweed, daphnia, and fish already indicate that DO has a risk factor of >1, which confirms the existence of a high risk to the aquatic environment [17]. Ir and Iy were calculated based on the number of duckweed fronds and frond area according to OECD 2006 guidelines [18]. In biotests over *E. crassipes*, a high concentration (15%) of DO caused a 2.73 cm decrease in the diameter of leaves after 6 days of exposure, which indicates the high degree of sensitivity of aquatic plants to oil pollution [9]. The inhibition of plant growth led to a noticeable decrease in the tissue mass of *L. minor*. As reported by Caja-Molina and Iannacone [17], the no-observed-effect concentration (NOEC) for *L. minor* was <0.4 mg L^−1^ for chlorosis and 3.22 mg L^−1^ for dry mass. Reduced wet weight indicates disruption of physiological processes, including nutrient uptake, photosynthesis, and cell division, making it a sensitive indicator of growth inhibition and toxic stress. Meanwhile, the Iy of *L. minor* exposed to the tested xenobiotic was more sensitive because this plant yielded an inhibition parameter that was higher than Ir (Figure 2).

Xenobiotics can disrupt the chlorophyll biosynthesis pathway [19], which leads to reduced photosynthetic activity and lower biomass productivity [20]. Fluorescence intensity varies depending on the duration and intensity of stress [21]. The highest Fo and Fm values of *P. subcapitata* in DO concentrations of 2.4% were significantly inhibited by 20.26% and 23%, compared to the control sample (Figure 3). The above photosynthetic indicators and the plant growth rate that decreased with increasing DO content in the nutrient solution suggest that DO is not neutral towards *P. subcapitata* algae. The reduced abundance of tested algae (characterized by high bioproductivity of producers) due to DO presence may be responsible for the inhibition of primary consumer survival in aquatic ecosystems. These studies suggest that algal chlorophyll fluorescence, particularly in Fo, Fm, and Fv measurements, is an early and sensitive indicator of the toxic effects of DO to algae before morphological changes are observed. The DO toxicity was reflected in Fv/Fm measurements. The Fv/Fm parameter is widely recognized as a reliable indicator of the photochemical activity of the photosynthetic apparatus. The Fv/Fm ratio reflects the efficiency of light energy utilization in the primary reactions of photosynthesis and is proportional to the quantum yield of photochemical reactions in photosystem II (PSII) [22]. In healthy leaves, the maximum quantum yield of PSII (Fv/Fm) ranges from 0.76 to 0.85 [23]. A decrease in Fv/Fm is commonly interpreted as a plant response to stress factors. Very low values of this parameter (0.2–0.3) indicate the occurrence of irreversible structural changes within the PSII complex [21]. Parallel analyses of photosynthetic processes, carried out using various measurement techniques, confirm that rapid measurements of chlorophyll fluorescence provide valuable information on the specific photosynthetic response of plants exposed to variable environmental factors [24]. The algae also responded with changes in the BA content when exposed to DO.

To date, the qualitative and quantitative profiles of BAs in the tissues of *P. subcapitata* have not been investigated, particularly under exposure to common environmental contaminants such as DO. This lack of data highlights a significant research gap concerning the metabolic responses of this species to xenobiotic stress. The analysis revealed the presence of several BAs in plant tissues, including monoamines (His, Tyr), diamines (Put, Cad, Agm), the triamine Spd, and the tetraamine Spm. Among all BAs, Agm was the most abundant in algal tissues, reaching 105.96 µg g^−1^ in controls. The DO exposure inhibited its biosynthesis, reducing the content to 47.67 µg g^−1^ at the highest concentration (Figure 4). The consequence of environmental stress on plant cells was the disruption of amine synthesis. Under stressful conditions, BAs can play a protective role for plants. This means that as environmental stress increases, the concentration of biogenic amines increases in plant tissues [25]. This relationship can be used to analyse the toxicity of chemical compounds by comparing the concentrations of amines secreted by an organism in a stress-free environment and in an environment contaminated with the chemical substance. Amine synthesis begins with decarboxylation—the enzymatic separation of the carboxyl group (-CO_2_) from the amino acid. The enzymes that channel this reaction are amino acid decarboxylases [26]. Agmatine is synthesized from L-arginine by arginine decarboxylase (ADC) and represents an important intermediate linking nitrogen metabolism with polyamine biosynthesis [27]. The DO reduced Agm levels in algae, most likely by inhibiting the enzyme ADC and favoring arginase activation. This increased Put levels, but not through activation of N-carbamoylputrescine. Increased Put levels during exposure to DO supported Spm levels indirectly. Although information on algae is more limited, available studies suggest that polyamine metabolism is also involved in the regulation of cell division, photosynthesis, and protection against oxidative damage induced by environmental stress [28]. Therefore, changes in Agm levels may reflect early metabolic adjustments to hydrocarbon-induced stress. However, the mechanisms regulating Agm metabolism in aquatic primary producers, particularly under diesel oil exposure, remain largely unexplored. As indicated in studies on *L. minor* dyes, it was ADC that was activated upon exposure to gentian violet [29]. In plants, the ADC pathway is activated under abiotic stress conditions, including salinity, drought, heavy metals, and oxidative stress, resulting in increased production of agmatine and downstream polyamines such as Put, Spd, and Spm [30]. A reduction in Spd biosynthesis by *L. minor* in response to a toxic agent was also observed in the study by Qiao et al. [31], where increasing cadmium concentrations led to a decrease in the content of this amine in the studied organisms.

Our experiment examining the effect of DO on *L. minor* showed that photosynthetic indicators Fo, Fm, and Fv/Fm decreased in the highest concentration of DO (2.4%) by 30, 45, and 8% (Figure 5, Table 5). The decrease in photosynthetic indices was the result of increasingly lower LCC in plants. While LCC increased by some percentage (24.92 SPAD) in the lowest DO concentration, the highest one decreased to the level of 15.22 SPAD (Figure 6). In vitro cultures of brown alga *Sargassum cymosum* exposed to DO at concentrations of 0.001%, 0.01%, 0.1% and 1%, *v*/*v* showed an increase in the content of chlorophyll a and c. Simultaneously, there was an increase in the thickness of the cell wall and the accumulation of phenolic compounds [8]. It was shown that in *L. minor* tissues, the content of Tyr, Put, Cad, Spd, and especially Agm (significant change in DO = 1.2%) increased in the presence of DO (Figure 7).

According to Chen et al. [28], BAs in living organisms occur mainly in free (F-PA), covalently conjugated (CC-PA), or non-covalently conjugated (NCC-PA) forms. In higher plants, BAs occur primarily in their free form. Free polyamines can form covalent amide bonds with low-molecular-weight compounds such as phenols and their derivatives. The phenolic compound involved in this reaction may include hydroxycinnamic acid, coumaric acid, caffeic acid, or ferulic acid. Recent studies using exogenous PAs, inhibitors of their biosynthesis, and transgenic techniques are intensively examining the role of polyamines in plant development and their mechanisms of action. These findings indicate that PAs play a key role in plant growth, the stability of nucleic acids and cell membrane structures, stress resistance, and overall plant survival. Harmful trace elements are introduced into the environment along with petroleum substances, and industrial activities in petrochemical plants lead to the accumulation of Cd, Cu, Cr, As, Zn, Pb, Ni, and Hg [32]. The application of the heavy metals Hg^2+^ and Cr^6+^ resulted in a reduction in the content of Spd and Spm and a decrease in the activity of antioxidant enzymes, superoxide dismutase (SOD), catalase, and peroxidase, in amaranth (*Brassica napus*) leaves. Consequently, excessive accumulation of lipid peroxides in cell membranes (malondialdehyde) and a significant reduction in the content of chlorophyll and soluble proteins were observed. Exogenous application of Spd mitigated the negative effects of the toxic effects of Hg^2+^ and Cr^6+^. Both *P. subcapitata* algae and duckweed (*L. minor*) responded to changes in the BA content in their tissues, but *L. minor* was generally less sensitive to the tested xenobiotic concentrations. The high sensitivity of the algae could be because *L. minor* plants floated on the surface of the medium, while the algae filled the entire sample volume. According to the literature, processes such as evaporation of volatile fractions, photochemical oxidation, mechanical grinding, and mixing with water contribute to weathering, thereby changing the chemical composition of DO over time. Lighter fractions may float away or volatilize, while heavier fractions remain in solution [33]. The toxicity of diesel oil is determined not only by physicochemical processes affecting hydrocarbon bioavailability but also by their specific modes of action in aquatic plants. Huang et al. [34] demonstrated that polycyclic aromatic hydrocarbons (PAHs) inhibited electron transport in photosystem I (PSI), followed by damage to photosystem II (PSII) in *Lemna gibba*, resulting in reduced carbon fixation and plant growth. Mallakin et al. [35] further showed that chlorophyll fluorescence detected hydrocarbon-induced impairment of the photosynthetic apparatus before visible toxicity symptoms appeared. Likewise, Marwood et al. [36] reported that exposure to PAH mixtures reduced PSII efficiency and inhibited the growth of *L. gibba*. Hydrocarbon toxicity has been reported for submerged macrophytes such as *Hydrilla verticillata*, where exposure resulted in growth inhibition, chlorophyll depletion, impaired photosynthetic performance, and oxidative stress [37]. Together with our findings, these studies indicate that disruption of photosynthetic electron transport, impairment of photosystem function, and oxidative stress are key mechanisms underlying the phytotoxicity of petroleum hydrocarbons in aquatic plants.

## 4. Materials and Methods

### 4.1. Chemicals

#### 4.1.1. Characteristics of the Tested Diesel Oil (DO)

Diesel oil (DO) is a commercial mixture of hydrocarbons (as a product: Ekodiesel ULTRA B, D, F, Class 2; EFECTA DIESEL B, D, F, VERVA ON B, D, F, Poland) [38]. The composition of the diesel oil used in the study is presented in Table 7.
Physical and chemical properties
Physical state:LiquidColour:Colourless or light yellowOdour:CharacteristicMelting point/freezing point:Not determinedBoiling point or initial boiling point and boiling range:175–180 °C—Initial boiling point 95% vol. distils  to 360 °CFlammability:Flammable liquid and vapourLower and upper explosion limit:No data (NOTE: Under specific conditions, product vapours may form explosive mixtures with air).Flash point:>56 °CAuto-ignition temperature:ca 240 °C [39]Decomposition temperature:Not applicable—mixturepH:Not applicableKinematic viscosity [40]2.0–4.5 mm^2^/s at 40 °C ca. 2.151 mm^2^/s at 50 °C For Class 2: 1.5–4.0 mm^2^/s at 40 °CSolubility:Insoluble in water; soluble in alcohols, hydrocarbons, ethers, carbon disulfide, carbon tetrachloride, chloroformPartition coefficient n-octanol/water (log value):Not applicable—mixtureVapour pressure:Not applicableDensity and/or relative density:0.820–0.845 g × cm^−3^ at 15 °CRelative vapour density:ca. 6 (air = 1)Particle characteristics:Not applicable

#### 4.1.2. Composition/Information on Ingredients

**Table 7:** Characteristics of diesel oil composition.

Substance Name	% Vol.	CAS No.
Fuels, diesel	83–100	68334-30-5
Petroleum gas oil fraction, co-processed with renewable hydrocarbons of plant and/or animal origin	0–10	Not applicable
Fatty acids, C16-18 and C18-unsatd., Me esters	0–7	67762-38-3
Fatty acids, vegetable oil, Me esters	0–7	68990-52-3
Fatty acids, C10-18 and C12-22-unsatd., C14-18 and C16-18-unsatd. alkyl esters	0–7	85049-31-6
Fatty acids, rape-oil, Me esters	0–7	85586-25-0

### 4.2. Algaltoxkit

The experiment utilized *P. subcapitata* plants purchased as part of the Algaltoxkit F kit (MicroBioTests Inc., Ghent, Belgium).

The toxicity of DO to *P. subcapitata* was assessed using the OECD algal growth inhibition test [12] and the ISO Water Quality-Freshwater Algal Growth Inhibition Tests with Unicellular Green Algae [41]. *P. subcapitata* (an algal density of 1.106 cells × mL^−1^) was grown in 10 cm path-length disposable long cells in polystyrene, containing 25 mL algae-toxicant dilutions in a plant growth chamber (ALL-Round-Al 185-4, Gent, Belgium) illuminated with fluorescent lights (140 µmol photon m^−2^ × s^−1^ PAR) in a light-to-dark cycle of 16 h/8 h (mean maximum temperature of 20 °C during daytime and 16 °C during nighttime) for 7 days [42]. All solutions were prepared using deionized water (Adrona Crystal 5 Basic water purification system, Riga, Latvia). Concentrations of commercial DO were prepared using growth medium. The responses of *P. subcapitata* to DO concentrations of 0, 0.3, 0.6, 1.2 and 2.4% (*m*/*v*) were determined based on the percent inhibition of growth rate (Ir) and the percent reduction in yield (Iy) of algae using optical density at a wavelength of 670 nm (Hitachi U-1800 spectrophotometer, Tokyo, Japan) according to the following equations:

*μ_i_*_–*j*_ = [ln(*N_j_*) − ln(*N_i_*)]/*t* (1)

*Ir* = (*μ_c_* − *μ_T_*)/*μ_c_* × 100 (2)

*Iy* = (*b_c_* − *b_T_*)/*b_c_* × 100 (3)

where *μ_i_* is the average specific growth rate in time *i* to *j*; *N_i_* is the measurement optical density in the test or control vessel at time *i*; *N_j_* is the measurement of optical density in the test or control vessel at time *j*; *T* is the period from *i* to *j*; *μ_c_* is the mean value of *µ* in the control group; *μ_T_* is the mean value of *µ* in the treatment group; *b_c_* is the final optical density minus the initial optical density in the control group; and *b_T_* is the final optical density minus the initial optical density in the treatment group.

### 4.3. Lemna Test

Experimental *L. minor* material was procured from the Department of Chemistry’s maintained plant nursery at the University of Warmia and Mazury in Olsztyn, Poland. *L. minor* was grown in 50 mL of OECD medium for testing chemicals [43] in a plant growth chamber (ALL–Round–Al 185–4, Gent, Belgium) illuminated with fluorescent light (140 μmol photon m^−2^ × s^−1^ PAR) in a 16 h light/8 h dark cycle (mean maximum temperature of 20 °C during the daytime and 16 °C during the nighttime) for 7 days. All solutions were prepared using deionized water (Adrona Crystal 5 Basic water purification system, Riga, Latvia). Concentrations of commercial DO were prepared using growth medium. The responses of common duckweed to all concentrations of the tested solutions (0, 0.3, 0.6, 1.2, 2.4% (*m*/*v*)) were determined based on the percent inhibition of growth rate (Ir), the percent reduction in yield (Iy), leaf greenness index (LG) of tissues, and Fv/Fm. The frond area was measured using the Lucia 5.0 program (Laboratory Imaging, s.r.o., Prague, Czech Republic). The following formulas were applied to calculate the values of Ir and Iy based on the number of duckweed fronds and frond area according to OECD 2006 guidelines [18].

*μ_i_*_–*j*_ = [ln(*N_j_*) − ln(*N_i_*)]/*t* (4)

*Ir* = (*μ_c_* − *μ_T_*)/*μ_c_* × 100 (5)

*Iy* = (*b_c_* − *b_T_*)/*b_c_* × 100 (6)

where *μ_i_* is the average specific growth rate in time *i* to *j*; *N_i_* is the measurement variable in the test or control vessel at time *i*; *N_j_* is the measurement variable in the test or control vessel at time *j*; *t* is the period from *i* to *j*; *μ_c_* is the mean value of *µ* in the control group; *μ_T_* is the mean value of *µ* in the treatment group; *b_c_* is the final number of duckweed fronds and frond area minus the initial number of duckweed fronds and frond area in the control group; and *b_T_* is the final number of duckweed fronds and frond area minus the initial number of duckweed fronds and frond area in the treatment group.

### 4.4. Leaf Greenness Index (LCC) of L. minor

The LCC was determined in *L. minor* plants using the SPAD 502 chlorophyll meter (Konica Minolta, Tokyo, Japan). It was expressed in unitless SPAD values.

### 4.5. Chlorophyll Fluorescence of P. subcapitata

The chlorophyll fluorescence of algae was measured with a HandyPEA chlorophyll fluorescence system equipped with a liquid-phase chlorophyll fluorescence adapter for Handy PEA (Hansatech Instruments Ltd., Pentney, UK). The algae-toxicant dilutions were transferred to 2 mL vials and stored in the dark for 30 min to quench chlorophyll fluorescence. After dark adaptation, chlorophyll was excited at a light intensity of 2500 [µmol × m^−2^ × s^−1^] to determine minimum chlorophyll fluorescence (Fo) and maximum chlorophyll fluorescence (Fm). The maximum quantum efficiency (Fv/Fm) of PSII (photosystem II) was determined based on the chlorophyll fluorescence kinetics of *P. subcapitata*.

### 4.6. Chlorophyll Fluorescence of L. minor

The chlorophyll fluorescence of *L. minor* Fv/Fm of PSII (photosystem II) was measured with the HandyPEA chlorophyll fluorescence system (Hansatech Instruments Ltd., Pentney, UK). Common duckweed leaves were placed in a leaf clip and stored in the dark for 30 min to quench chlorophyll fluorescence. After dark adaptation, chlorophyll was excited at a light intensity of 2500 [µmol × m^−2^ × s^−1^], and minimum chlorophyll fluorescence (Fo), maximum chlorophyll fluorescence (Fm), and variable chlorophyll fluorescence (Fv = Fm − Fo) were determined. The Fv/Fm of PSII was determined based on the chlorophyll fluorescence kinetics of common duckweed.

### 4.7. Biogenic Amines in P. subcapitata and L. minor Tissues

Biogenic amines (BAs), crucial biochemical compounds found in plant material (min. 0.5 g), were extracted on ice using 5% hydrochloric acid at 4 °C. This method ensured the precise isolation and preservation of these biologically significant substances for subsequent analytical processes and research investigations [44,45,46]. Following extraction, the plant material underwent agitation for 1 h at 1500 rpm using Multi-Vortex V-32 (Biosan, Riga, Latvia), followed by centrifugation at 16,000× *g* for 30 min at a temperature of 4 °C in centrifuge MPW-150R (MPW Med. Instruments, Warsaw, Poland) to facilitate the separation of components. The resulting supernatants were filtered through a 0.22 µm pore nylon membrane syringe filter (Filter-Bio, Nantong, China) and subsequently preserved at −20 °C to maintain their integrity. The filtrate underwent thorough analysis via ion-exchange chromatography utilizing the AAA400 amino acid analyser (INGOS Ltd., Prague, Czech Republic). The buffer system, analytical protocols (including elution programs), and preparation of the ninhydrin reagent were implemented according to the manufacturer’s instructions (INGOS Ltd., Prague, Czech Republic). Within this process, BAs were separated at a temperature of 76 °C, employing a 70 × 3.7 mm column packed with POLY 8 (INGOS Ltd., Prague, Czech Republic). Elution from the ion-exchange column was achieved through two distinct sodium citrate buffers. Buffer A contained 5.5 mM citric acid, 81 mM sodium citrate, 257 mM NaCl, 350 mM KBr, and 250 mL of isopropanol, with a final pH of 5.78. Buffer B contained 73 mM citric acid, 3 M NaCl, and 10 mL of 50% KOH, with a final pH of 3.27 (WTW inoLab pH meter, Xylem Analytics Germany, Weilheim, Germany). Quantitative and qualitative assessments of the BAs were conducted through post-column ninhydrin derivatization (sensitivity: <50 pmol (S/N = 5), sample volume injected: 100 µL, flow cell volume: standard 5 µL, ninhydrin reactor: ambient −150 °C) coupled with photometric detection (λ = 570 nm) (INGOS Ltd., Prague, Czech Republic). Chromatographic analysis utilized BA standards provided by Sigma Aldrich (Saint Louis, MO, USA) to ensure accuracy and reliability. The number of BAs was expressed as the mean ± standard deviation for 6 replicates in each treatment, ensuring robust statistical analysis and interpretation of results. The method for the determination of BAs was validated by analysing known concentrations of external standards of these substances (Table S5) [47].

### 4.8. Statistical Analysis

The experiment was conducted in six replicates. The results were expressed as means ± standard deviation (SD). Data were processed statistically by one- and two-way (only for Ir and Iy) analysis of variance (ANOVA) (F test) (Tables S1–S4). The experimental factors were the time and concentration of the applied DO. Significant differences were determined using Tukey’s test at *p* < 0.01. The results of the experiment were processed in the STATISTICA 13.3 statistical package (TIBCO Software Inc., Palo Alto, CA, USA). Effective concentrations (EC_x_) were analysed with a selected regression model to calculate the concentrations at 20% and 50% response levels. Effective concentration (ECx) data were analysed separately for each replicate using a plot and equation of the dependence of the response (morphological and physiological features) of organisms (% of control) on the logarithm of the test substance’s concentration to calculate the concentrations at 20%, 50%, and 90% response levels. For the values, the mean (m) and 95% confidence intervals (*CI*) were determined using Student’s t-distribution (*α* = 0.05).

## 5. Conclusions

This study concluded that DO pollution is a serious environmental issue that has a variety of severe impacts on the aquatic environment. DO—by altering tested several plant characteristics, including growth, biomass, and productivity—and the environmental effects of petroleum activities may have an impact on specific species, including producers, and then on populations, assemblages, or ecosystems. As indicated in the experiment, petroleum contamination in aquatic ecosystems has short-term impacts too. Attention has been paid to the issues posed by point pollution in aquatic ecosystems due to the impact of DO and its depleting effects on the morphological and physiological aspects of aquatic plants like algae *P. subcapitata* and the floating plant *L. minor*. The results of this study confirm that increasing concentrations of DO adversely affect the morphological and physiological parameters of *P. subcapitata* and *L. minor*. It should be noted that *L. minor* (directly exposed to DO) was generally less sensitive to the tested xenobiotic concentrations in relation to its growth characteristics. The calculated toxicity rates indicate that DO concentrations exceeding 0.11% and 0.12% (LOEC after 7 days) were phytotoxic, impairing the growth and yield of these model organisms used in biological research. The measured photosynthetic indices, which decreased with increasing DO content in the nutrient solution, indicate that DO is not neutral to the studied aquatic plants. Consequently, reduced plant productivity may be responsible for inhibiting the survival of primary consumers in aquatic ecosystems. These studies suggest that the chlorophyll fluorescence metrics tested in the experiment, especially the Fo and Fm measurements, are early and sensitive indicators of the toxic effects of DO on *P. subcapitata* and *L. minor*, before morphological changes are observed.

Concurrently, alterations in BA levels within indicator plant tissues were observed as a result of DO exposure. In this study, the biogenic amine (BA) profile in the cells of the green alga *P. subcapitata* was assessed for the first time. Algal tissues contain His, Tyr, Put, Cad, Agm, Spd, and Spm. The obtained results allow us to conclude that Agm is a common indicator of DO water contamination for the two tested organisms; however, its concentration increased in algae, while in duckweed it decreased. In the next stage, to consider Agm as a universal biomarker, emphasis should be placed on determining the activity of arginine decarboxylase and tracing the biosynthesis pathway of all BAs. It was found that the content of Agm of *P. subcapitata* decreased at an EC_50_ of DO = 0.85% and increased in *L. minor* at an EC_50_ of 0.38%. The results of this study also confirm that the Algaltoxkit biological method and the Lemna test are useful analytical tools for assessing the toxicity of diesel oil and predicting the effects of the pollution of surface freshwater reservoirs with petroleum derivatives.

## Figures and Tables

**Table 1:** The effect of diesel oil (DO) on morphological indices: percent inhibition of growth rate (Ir) and percent inhibition in yield (Iy) of *Pseudokirchneriella subcapitata*. The table below contains the mean (m) and 95% confidence intervals (*CI*) for the mean.

Feature		Diesel Oil [%]			
			EC_20_	EC_50_	EC_90_
**3 day**	Percent inhibition of growth rate (Ir), %		0.54	0.71	1.29
		*CI*	0.43 < m < 0.65	0.58 < m < 0.83	1.04 < m < 1.54
	Percent inhibition in yield (Iy), %		0.25	0.40	1.28
		*CI*	0.06 < m < 0.44	0.38 < m < 0.41	0.99 < m < 1.57
**5 day**	Percent inhibition of growth rate (Ir), %		0.22	0.35	1.02
		*CI*	0.13 < m < 0.30	0.26 < m < 0.45	0.64 < m < 1.40
	Percent inhibition in yield (Iy), %		0.16	0.32	0.64
		*CI*	0.10 < m < 0.23	0.24 < m < 0.40	0.35 < m < 0.92
**7 day**	Percent inhibition of growth rate (Ir), %		0.08	0.19	0.39
		*CI*	0.07 < m < 0.08	0.18 < m < 0.21	0.21 < m < 0.56
	Percent inhibition in yield (Iy), %		0.14	0.26	0.43
		*CI*	0.08 < m < 0.20	0.21 < m < 0.32	0.24 < m < 0.62

**Table 2:** The effect of diesel oil (DO) on morphological indices: percent inhibition of growth rate (Ir), percent inhibition in yield (Iy) of *Lemna minor*. The table contains the mean (m) and 95% confidence intervals (*CI*) for the mean.

Feature		Diesel Oil [%]			
			EC_20_	EC_50_	EC_90_
**3 day**	Percent inhibition of growth rate (Ir), %		0.20	0.46	-
		*CI*	0.15 < m < 0.24	0.25 < m < 0.66	-
	Percent inhibition in yield (Iy), %		0.14	0.51	-
		*CI*	0.13 < m < 0.16	0.35 < m < 0.68	-
**5 day**	Percent inhibition of growth rate (Ir), %		0.14	0.35	-
		*CI*	0.13 < m < 0.14	0.34 < m < 0.35	-
	Percent inhibition in yield (Iy), %		0.10	0.26	-
		*CI*	0.10 < m < 0.10	0.26 < m < 0.26	-
**7 day**	Percent inhibition of growth rate (Ir), %		0.14	0.40	-
		*CI*	0.13 < m < 0.16	0.34 < m < 0.46	-
	Percent inhibition in yield (Iy), %		0.10	0.26	-
		*CI*	0.09 < m < 0.11	0.24 < m < 0.28	-

**Table 3:** The alga *Pseudokirchneriella subcapitata* exposed to 0–2.40% of diesel oil (DO) and the effect of DO on maximum quantum efficiency (Fv/Fm) of PSII (photosystem II) of plants. Data points represent the mean ±SD, *n* = 6. * Values differ significantly from the control at *p* < 0.01.

Diesel Oil [%]	Maximum Photochemical Efficiency PSII (Fv/Fm); Mean ± SD	Symptoms
**0.00**	0.66 * ± 0.01 *	** 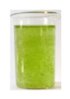 **
**0.30**	0.69 * ± 0.00 *	** 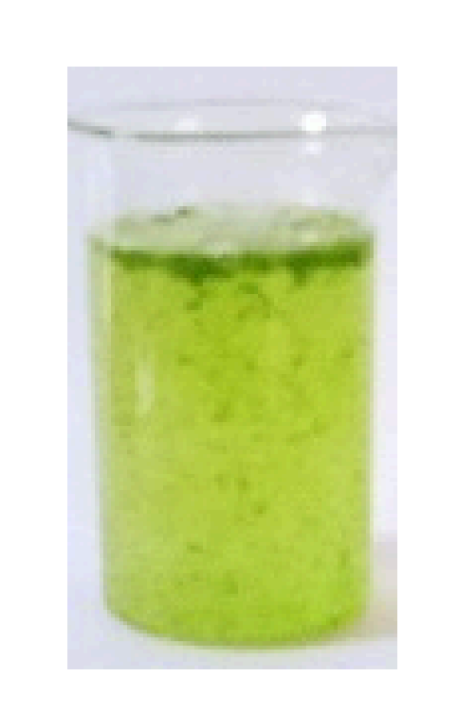 **
**0.60**	0.69 * ± 0.00 *	** 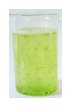 **
**1.20**	0.68 * ± 0.00 *	** 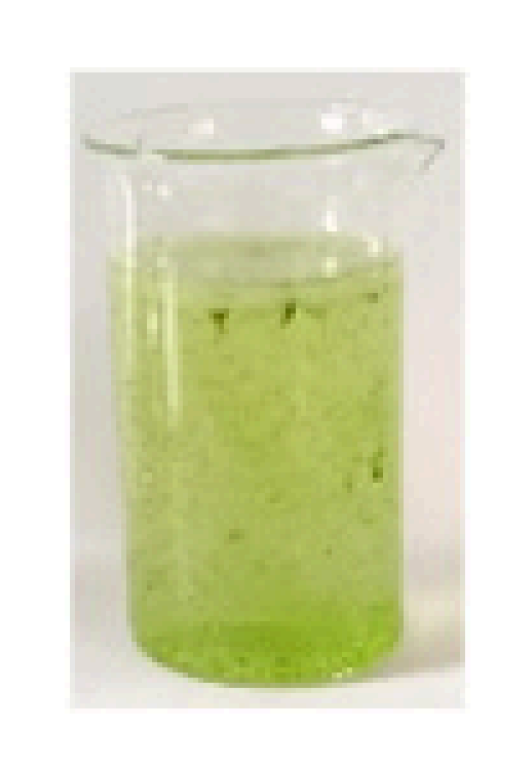 **
**2.40**	0.68 * ± 0.01 *	** 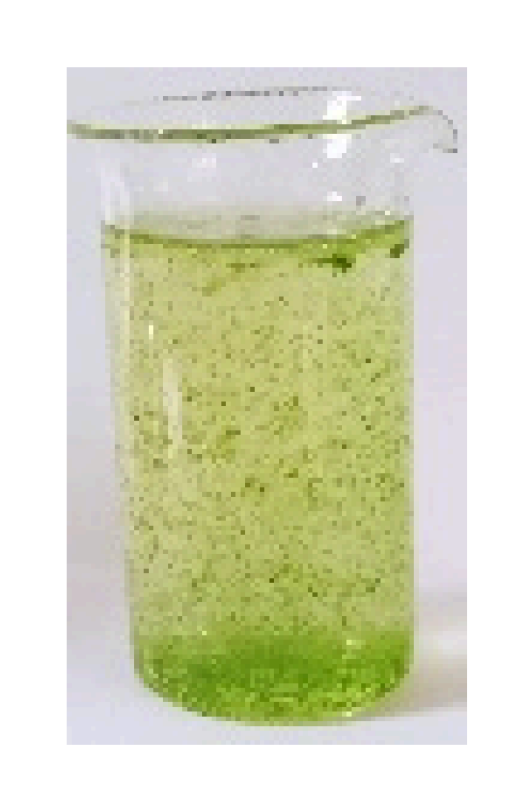 **

**Table 4:** The effect of diesel oil (DO) on physiological parameters/features of algae *Pseudokirchneriella subcapitata*. The table below contains the mean (m) and 95% confidence intervals (*CI*) for the mean.

Feature		Diesel Oil [%]		
		EC_20_	EC_50_	EC_90_
Min. chlorophyll fluorescence (Fo)		2.30	-	-
	*CI*	2.09 < m < 2.38	-	-
Max. chlorophyll fluorescence (Fm)		2.14	-	-
	*CI*	2.08 < m < 2.21	-	-
Max. quantum efficiency (Fv/Fm)		-	-	-
	*CI*	-	-	-
Histamine content (His)		0.22	0.42	-
	*CI*	0.16 < m < 0.29	0.38 < m < 0.45	-
Tyramine content (Tyr)		0.04	0.11	0.20
	*CI*	0.04 < m < 0.05	0.10 < m < 0.12	0.19 < m < 0.22
Putrescine content (Put)		0.03	0.08	0.15
	*CI*	0.03 < m < 0.03	0.08 < m < 0.09	0.15 < m < 0.16
Cadaverine content (Cad)		0.18	0.44	-
	*CI*	0.15 < m < 0.21	0.40 < m < 0.48	-
Spermidine content (Spd)		0.16	0.47	-
	*CI*	0.15 < m < 0.17	0.44 < m < 0.50	-
Spermine content (Spm)		0.06	0.14	0.26
	*CI*	0.05 < m < 0.06	0.13 < m < 0.16	0.23 < m < 0.29
Agmatine content (Agm)		0.27	0.85	-
	*CI*	0.22 < m < 0.31	0.78 < m < 0.93	-

**Table 5:** *Lemna minor* plants exposed to 0–2.40% of diesel oil (DO) and the effect of DO on maximum quantum efficiency (Fv/Fm) of the PSII (photosystem II) of plants. Data points represent the mean ±SD, *n* = 6.

Diesel Oil [%]	Maximum Photochemical Efficiency PSII (Fv/Fm); Mean ± SD	Symptoms
**0.00**	0.79 ± 0.01	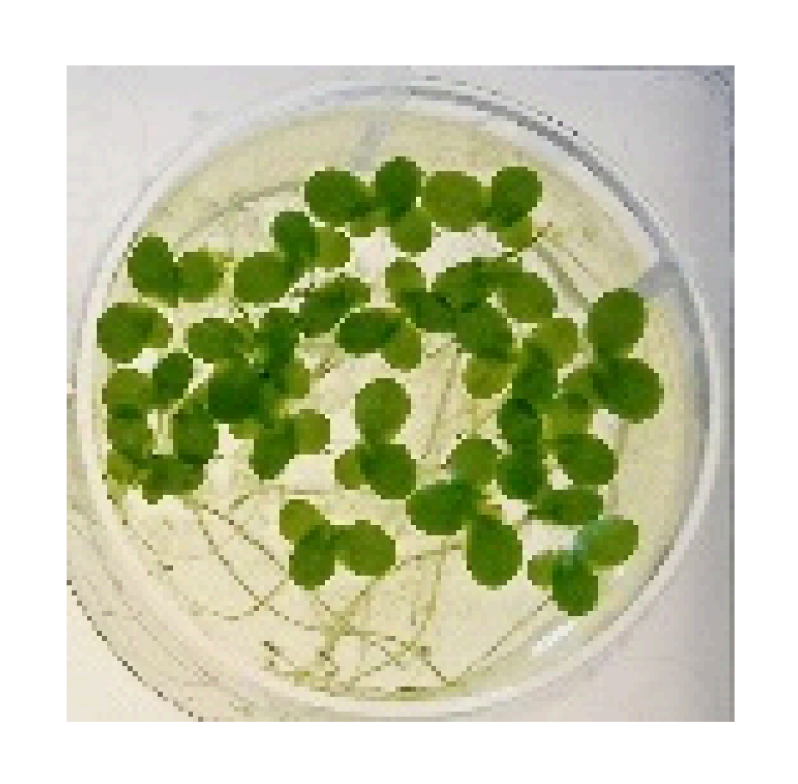
**0.30**	0.79 ± 0.01	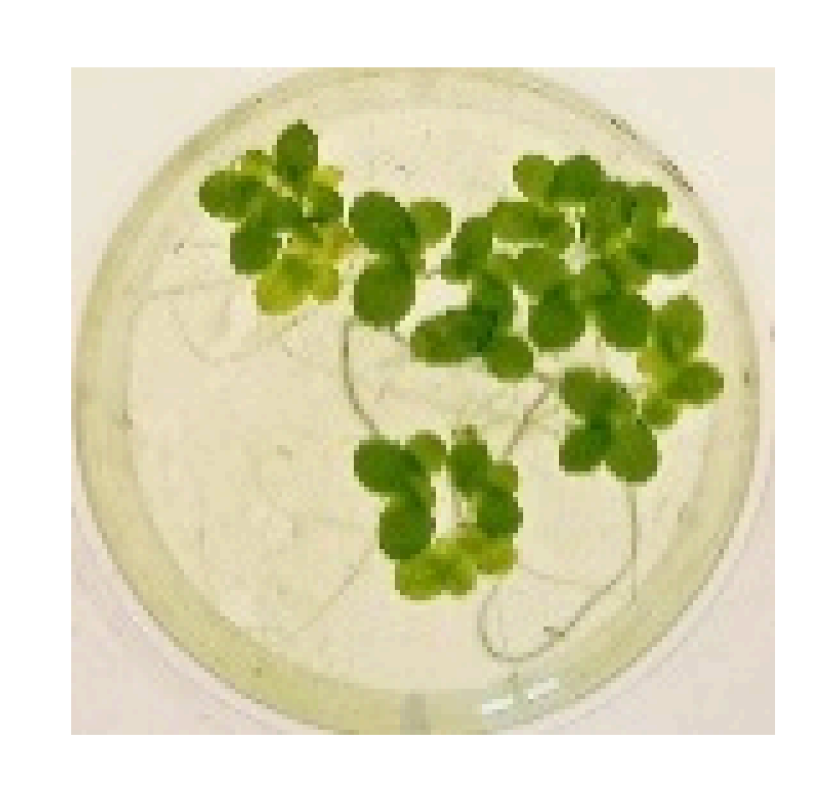
**0.60**	0.79 ± 0.01	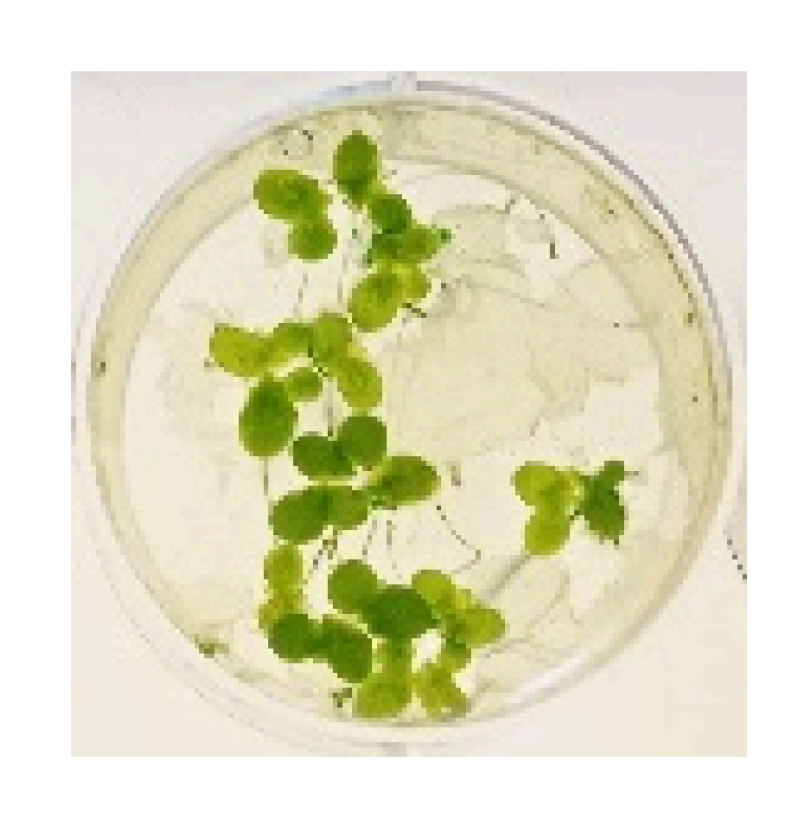
**1.20**	0.78 ± 0.02	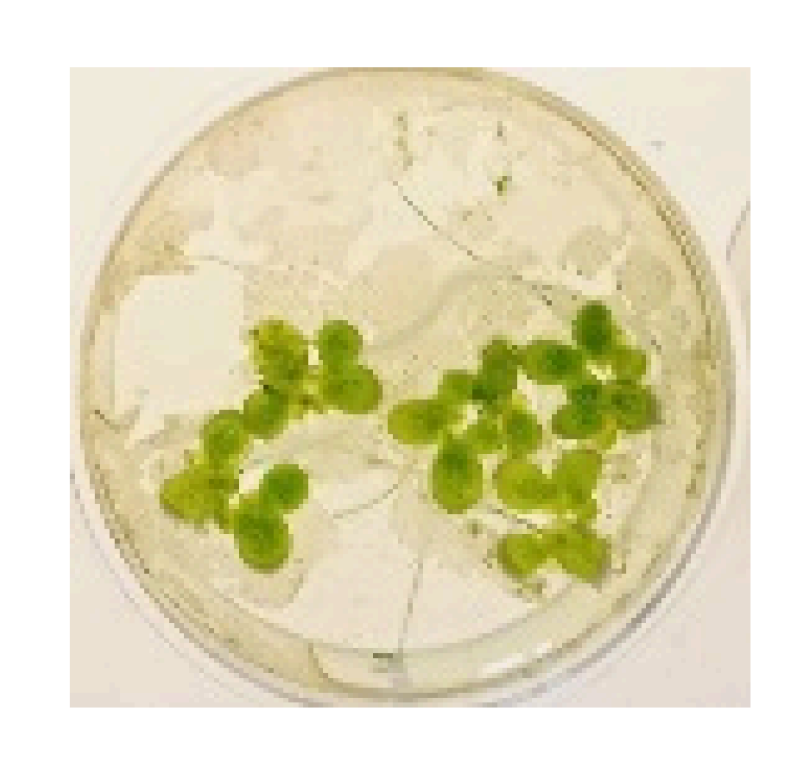
**2.40**	0.73 ± 0.04	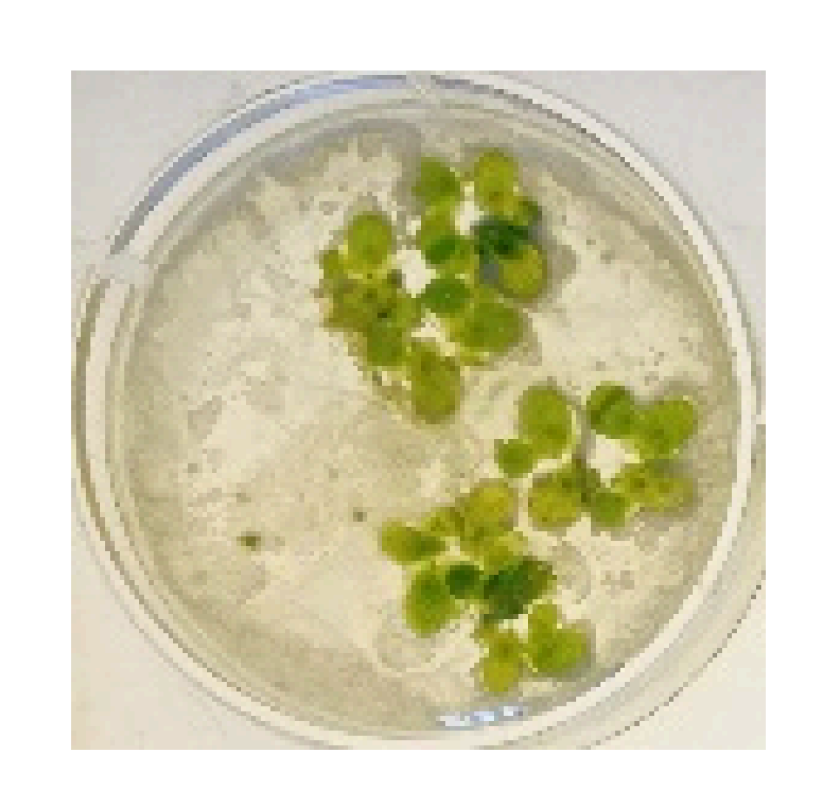

**Table 6:** The effect of diesel oil (DO) on physiological parameters/features of *Lemna minor* plants. The table below contains the mean (m) and 95% confidence intervals (*CI*) for the mean.

Feature	Diesel Oil [%]			
		EC_20_	EC_50_	EC_90_
Leaf chlorophyll content (LCC)		1.00	-	-
	*CI*	0.73 < m < 1.26	-	-
Min. chlorophyll fluorescence (Fo)		0.55	-	-
	*CI*	0.48 < m < 0.63	-	-
Max. chlorophyll fluorescence (Fm)		0.43	-	-
	*CI*	0.37 < m < 0.49	-	-
Max. quantum efficiency (Fv/Fm)		-	-	-
	*CI*	-	-	-
Tyramine content (Tyr)		0.89	0.97	-
	*CI*	0.78 < m < 0.99	0.53 < m < 1.41	-
Putrescine content (Put)		0.81	0.93	-
	*CI*	0.70 < m < 0.93	0.58 < m < 1.29	-
Cadaverine content (Cad)		0.32	0.49	0.71
	*CI*	0.20 < m < 0.43	0.39 < m < 0.58	0.63 < m < 0.79
Spermidine content (Spd)		0.07	0.19	0.24
	*CI*	0.05 < m < 0.10	0.13 < m < 0.25	0.13 < m < 0.35
Spermine content (Spm)		-	-	-
	*CI*	-	-	-
Agmatine content (Agm)		0.30	0.38	0.45
	*CI*	0.23 < m < 0.36	0.34 < m < 0.42	0.40 < m < 0.49

## Data Availability

The datasets generated during and/or analysed during the current study are available from the corresponding author upon reasonable request.

## Supplementary Materials

The following supporting information can be downloaded at https://www.mdpi.com/article/10.3390/molecules31162838/s1, Table S1: Analysis of variance (ANOVA) of the morphological indices of algae Pseudokirchneriella subcapitata exposed to different concentrations (0–2.40%) of diesel oil (DO); Table S2: Analysis of variance (ANOVA) of the morphological indices of Lemna minor exposed to different concentrations (0–2.40%) of diesel oil (DO); Table S3. Analysis of variance (ANOVA) of the physiological parameters/features of algae Pseudokirchneriella subcapitata exposed to different concentrations (0–2.40%) of diesel oil (DO); Table S4. Analysis of variance (ANOVA) of the physiological parameters/features of *Lemna minor* plants exposed to different concentrations (0–2.40%) of diesel oil (DO); Table S5. Analytical parameters of AAA-400.

## Abbreviations

The following abbreviations are used in this manuscript:

DO	diesel oil
Ir	percent inhibition of growth rate
Iy	percent reduction in yield
Fo	minimum chlorophyll fluorescence
Fm	maximum chlorophyll fluorescence
Fv/Fm	maximum quantum efficiency
LCC	leaf chlorophyll content
EC_x_	effective concentration associated with x% response (20% and 50%)
LOEC	lowest observed effect concentration
BAs	biogenic amines
His	histamine
Tyr	tyramine
Put	putrescine
Cad	cadaverine
Spd	spermidine
Agm	agmatine
Spm	spermine
