# Supplementary material for: Biogenic Amines as Biomarkers for the Assessment of Diesel-Contaminated Water Toxicity
Source: Molecules. 2026 Aug 14;31(16):2838. doi: 10.3390/molecules31162838 (PMC13515937; doi:10.3390/molecules31162838)
Supplement: Supplementary file 1 [file molecules-31-02838-s001.zip › molecules-4470707-supplementary.pdf]

Supplementary

Table S1. Analysis of variance (ANOVA) of the morphological indices of algae *Pseudokirchneriella subcapitata* exposed to different concentrations (0–2.40%) of diesel oil (DO).

Source of Variation	Ir	Iy
	F-value	
Intercept	296.12 *	124.43 *
Concentration (C)	33.12 *	15.17 *
Time (T)	5.08 *	3.14
C × T	1.34	0.48

C—concentration, T—time, C × T—interactions between the factors, * significant at $p < 0.01$.

Table S2. Analysis of variance (ANOVA) of the morphological indices of *Lemna minor* exposed to different concentrations (0–2.40%) of diesel oil (DO).

Source of Variation	Ir	Iy
	F-value	
Intercept	657.65 *	1324.67 *
Concentration (C)	45.03 *	87.15 *
Time (T)	2.71	8.22 *
C × T	0.34	0.66

C—concentration, T—time, C × T—interactions between the factors, * significant at $p < 0.01$.

Table S3. Analysis of variance (ANOVA) of the physiological parameters/features of algae *Pseudokirchneriella subcapitata* exposed to different concentrations (0–2.40%) of diesel oil (DO).

SoV	Fo	Fm	Fv/Fm	His	Tyr	Put	Cad	Spd	Spm	Agm
					F-value					
Intercept	2462.52*	2276.72*	179,788.30 *	686.37*	463.00*	595.24*	900.02*	1381.82*	775.04*	3724.92*
Concentration (C)	4.38*	5.41*	10.45*	33.24*	17.35*	15.11*	88.38*	67.75*	13.43*	102.36*

SoV—Source of variation, C—concentration, * significant at $p < 0.01$.

Table S4. Analysis of variance (ANOVA) of the physiological parameters/features of *Lemna minor* plants exposed to different concentrations (0–2.40%) of diesel oil (DO).

SoV	Fo	Fm	Fv/Fm	LCC	Tyr	Put	Cad	Spd	Spm	Agm
					F-value					
Intercept	1301.99*	864.08*	15,597.00 *	1115.45*	21.73*	22.86*	13.42*	157.11*	88.05*	49.96*
Concentration (C)	8.97*	10.53*	3.42	6.57*	0.50	0.87	1.21	1.72	0.25	4.86*

SoV—Source of variation, C—concentration, * significant at $p < 0.01$.

Table S5. Analytical parameters of AAA-400.

Biogenic Amine	Molar Mass [g × mol⁻¹]	Structural Formula	Regression Equation	Retention Time [min]	R^2	LOD [µg × 100 µL⁻¹]	LOQ [µg × 100 µL⁻¹]	RSD [%]
Histamine	111.15	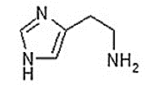	$y = 4 \times 10^6$	19	0.9987	0.26	27.83	8.22
Tyramine	137.18	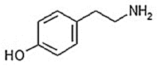	$y = 4 \times 10^6$	27	0.9974	1.02	33.30	5.30
Putrescine	88.15	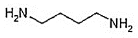	$y = 1 \times 10^7$	41	0.9982	0.22	22.04	9.19
Cadaverine	102.18	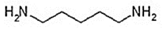	$y = 6 \times 10^6$	46	0.9983	0.25	25.30	5.47
Spermidine	145.25	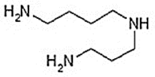	$y = 9 \times 10^6$	54	0.9944	0.23	24.88	12.12
Agmatine	130.19	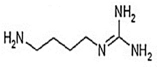	$y = 3 \times 10^6$	59	0.9964	0.33	32.55	7.93
Spermine	202.34	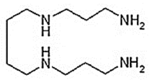	$y = 8 \times 10^6$	66	1.0000	0.60	50.59	2.27

R^2—regression coefficient, LOD—limit of detection, LOQ—limit of quantification, RSD—relative standard deviation.
